# The clinical application and challenges of preimplantation genetic testing

**DOI:** 10.3389/fgene.2025.1599088

**Published:** 2025-06-02

**Authors:** Fan Zhou, Xinlian Chen, Shanling Liu, Xiaodong Wang

**Affiliations:** ^1^ Department of Medical Genetics/Prenatal Diagnostic Center, West China Second University Hospital, Sichuan University, Chengdu, Sichuan, China; ^2^ Key Laboratory of Birth Defects and Related Diseases of Women and Children (Sichuan University), Ministry of Education, Chengdu, Sichuan, China; ^3^ Department of Obstetrics and Gynecology, West China Second University Hospital, Sichuan University, Chengdu, Sichuan, China

**Keywords:** preimplantation genetic testing, chromosomal structural rearrangement, monogenic disease, aneuploidies, clinical application, challenges

## Abstract

Preimplantation genetic testing (PGT) has rapidly advanced due to the significant development of genetic testing technologies. As an integration of genetic testing and assisted reproductive technology (ART), PGT plays a pivotal role in the primary prevention of birth defects, mainly chromosomal abnormalities and monogenic disease with known pathogenic variants. Blastocyst biopsy entails the collection of a relatively higher number of cells compared to other methods. Thereafter, whole genome amplification (WGA) generates a substantially larger amount of DNA templates, enabling more accurate subsequent genetic analyses. As an evolving technique that continues to be improved, the inherent limitations of WGA are expected to be minimized in the near future. Despite the widespread application of genetic techniques to WGA products, challenges remain in the downstream detection of small-fragment copy number variations (CNVs) (particularly those <1 Mb), the inability of long-read sequencing to resolve haplotypes or determine the position and orientation of micro-duplications for specific genomic sequences. Additionally, identifying complex or cryptic structures of balanced chromosomal rearrangements in prospective parents with a history of adverse pregnancy outcomes represents an urgent and challenging task, which would facilitate the pre-testing evaluation of PGT indications. Meanwhile, further assessment of the risks associated with transferring embryos with mosaic chromosome abnormalities, the implantation potential of euploid embryos, as well as the long-term health outcomes of children born following PGT requires more rigorously designed studies to provide robust evidence. The technology of PGT will continue to evolve, becoming increasingly comprehensive and precise. However, this technology should be applied strictly in accordance with legislation and ethical guidelines, with the ultimate aim of benefiting couples.

## Introduction

**FIGURE 1:**
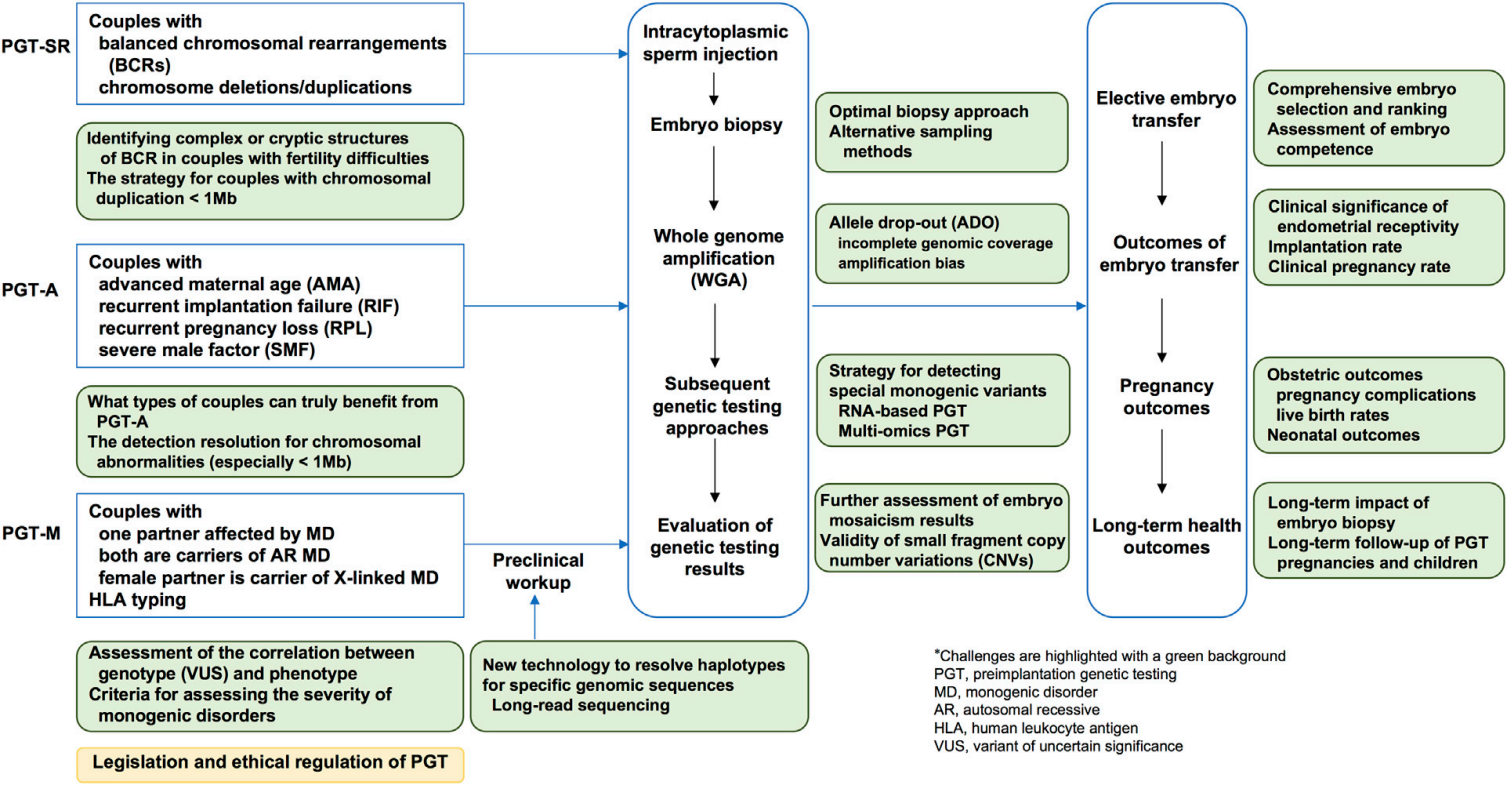
A schematic illustration of the clinical applications and challenges of preimplantation genetic testing.

The field of preimplantation genetic testing (PGT) has rapidly evolved over the past 2 decades due to the development of new genetic testing technologies. PGT integrates genetic testing with assisted reproductive technology (ART) and is categorized into three types based on specific clinical indications: PGT for chromosomal structural rearrangements (PGT-SR), PGT for monogenic diseases (PGT-M), and PGT for aneuploidies (PGT-A). Chromosomal structural rearrangements, including reciprocal translocations, Robertsonian translocations, insertional translocations, and inversions, constitute a major indication for PGT-SR (ESHRE PGT-SR/PGT-A Working Group et al., 2020). PGT-M involves testing for monogenic disorders caused by pathogenic variants in nuclear DNA, with an autosomal dominant, autosomal recessive, or X-linked inheritance patterns. It also encompasses mitochondrial DNA pathogenic variant detection and human leukocyte antigen (HLA) typing (ESHRE PGT-M Working Group et al., 2020). Detailed indications for PGT-M include cases in which one of the couple is affected by a monogenic disorder or carries mutations in high-penetrance susceptibility genes that lead to a genetic predisposition for severe phenotypes, both husband and wife are carriers of the same monogenic disorder with an autosome recessive inheritance pattern, or the female partner is a carrier of a monogenic disorder with an X-linked inheritance pattern (Yan et al., 2023). PGT-A is indicated for couples with advanced maternal age (AMA), recurrent implantation failure (RIF), severe male factor (SMF) infertility, or those couples with normal karyotypes who have experienced recurrent pregnancy loss (ESHRE PGT-SR/PGT-A Working Group et al., 2020). Importantly, contraindications for PGT should be considered, including diseases with unidentified causative genes, non-medical embryo selection for non-disease phenotypes, contraindications related to pregnancy or assisted reproductive technology, and cases not permitted by local laws or not approved by relevant medical ethics committees (Yan et al., 2023). A series of guidelines and committee opinions on the application of PGT technology in clinical practice have been published (Verdonschot et al., 2024; Preimplantation Genetic Testing: ACOG Committee Opinion, 2020; Practice Committees of the American Society for Reproductive Medicine and the Society for Assisted Reproductive Technology, 2024). While PGT plays an important role in the primary prevention of birth defects, it also faces enormous challenges. This review focusses on recent developments and emerging evidence relevant to the clinical application of PGT, as well as future efforts required in both academic and clinical research in this field (Figure 1).

## Whole-genome amplification (WGA)

One of the most significant challenges across all categories of PGT arise from the limited amount of input DNA. Typically, a single biopsied polar body (PB), a single blastomere cell, or 5–10 trophectoderm (TE) cells undergo whole-genome amplification (WGA) step to generate relative larger amount of DNA for subsequent analysis (ESHRE PGT Consortium and SIG-Embryology Biopsy Working Group et al., 2020). Blastocyst biopsy entails the collection of a relatively higher number of cells and offers several advantages over alternative biopsy procedures. Ideally, the WGA procedure should ensure high genomic coverage, preserve the inherent sequence composition without introducing artificial sequence variation or causing artificial loss of gene copies, and enable reliable quantification of copy number variations (Czyz et al., 2015). The primary WGA strategies used in PGT include degenerate oligonucleotide primer PCR (DOP-PCR), multiple displacement amplification (MDA), multiple annealing and looping-based amplification cycles (MALBAC), and Picoplex, a hybrid WGA technique. Allele drop-out (ADO) is defined as the situation where one of two alleles in a heterozygous sample is amplified while the other remains undetected. ADO results from incomplete genomic coverage or amplification bias (preferential amplification of one of the alleles) and is widely recognized as an inherent limitation of WGA products derived from blastocyst-biopsied cells in PGT. ADO severely impacts the reliability of diagnostic results and poses challenges to the analysis of single nucleotide variants (SNVs) and copy number variations (CNVs) in embryonic genomes. Furthermore, each WGA approach has its own strengths and limitations, and ADO is influenced by the specific molecular technique employed (Volozonoka et al., 2022). According to ESHRE guidelines, MDA is recommended for identifying SNVs in PGT-M, while DOP-PCR (also known as Picoplex/Sureplex) is suggested as the preferred method for detecting CNVs (ESHRE PGT-M Working Group et al., 2020). It has been reported that Picoplex is currently the most widely adopted solution for PGT-A when using either array-based or next-generation sequencing (NGS)-based platforms. As WGA is an evolving technique that continues to be improved, many of the limitations associated with current methods are expected to be minimized in the near future.

## Clinical application of PGT-SR and challenges

Chromosomal structural rearrangements are categorized into balanced chromosomal rearrangements (BCRs), chromosome deletions, and chromosome duplications. BCRs are widely recognized causes of infertility, recurrent miscarriage in natural conception, recurrent implantation failure (RIF) in assisted reproductive technology, and even birth defects or developmental delays in offspring. Peripheral blood karyotype analysis serves as the first-line diagnostic approach for identifying BCRs in prospective parents, including reciprocal translocations, Robertsonian translocations, inversions, insertions, and complex BCRs involving three or more chromosomes or featuring three or more breakpoints. Population-based studies have reported that the incidence of BCRs ranges from 1 in 560 for reciprocal translocations to 1 in 1,100–1,200 for inversions and Robertsonian translocations (Forabosco et al., 2009). Comprehensive recommendations on the technical aspects of PGT-SR are outlined in the ESHRE guidelines on good practice for PGT (ESHRE PGT-SR/PGT-A Working Group et al., 2020).

For couples with BCRs who choose PGT-SR, preventing the transfer of blastocysts with translocation-related chromosomal abnormalities or aneuploidies can help shorten the time-to-pregnancy and minimize the risk of spontaneous or induced abortion due to fetal anomalies, on the basis of not increasing adverse fetal outcomes through embryo diagnosis and elective embryo transfer (ESHRE PGT-SR/PGT-A Working Group et al., 2020; Cimadomo et al., 2023; Benn and Merrion, 2024). Evidence shown that the likelihood of obtaining a live birth in couples with BCRs is influenced by the subtype of BCR, specific chromosomes involved in rearrangement, gender of BCR carrier, maternal age, and history of recurrent miscarriage (Verdoni et al., 2021; Nakano et al., 2022). Consequently, the possibility of having a live birth in both natural conception and PGT-SR showed large variability among couples carrying BCRs. In couples with BCRs, the overall euploidy rate of blastocysts has been reported to range from 34.02% to 35.29%, with the highest rate observed in inversion (57.27%), followed by Robertsonian translocations (46.06%) and reciprocal translocations (30.11%) (Zhou et al., 2024; Zhang et al., 2025). A systematic review of PGT-A and PGT-SR revealed a live birth rate (LBR) of 26.7%–87% among 562 couples who underwent PGT-SR, compared to 25%–71% among 847 couples who conceived naturally (Iews et al., 2018). However, the intrinsic risks and chances of obtaining a live birth through PGT-SR or natural conception in BCR carrier couples remain to be understand. For couples firstly diagnosed with BCRs, the choice of natural conception or assisted reproductive technology is challenging for both the couples and healthcare providers. While PGT may be partly effective for implantation in infertility patients, reduce miscarriage rates and shorten the time required to achieve a live birth, it also entails a high financial burden and potential risks related to invasive procedures. Given that couples with BCRs also have alternative options, such as assisted reproduction using donor semen in cases of male BCR carriers, or even adoption, it is crucial to further explore which features are associated with the reproductive competence of BCR carrier couples. This evidence is essential to define, implement, and validate safer and more efficient clinical workflows. More data on the pregnancy outcomes of couples with BCRs choosing PGT or natural conception are needed to provide further evidence on the genetic counselling and to guide reproductive decision-making.

More importantly, identifying the chromosome rearrangements in couples is the first step of evaluating indications for PGT-SR. While traditional karyotype analysis is unable to detect chromosome translocations with fragment sizes smaller than 5 Mb, as well as complex or cryptic structures (Hardisty and Vora, 2014), additional technologies have applied to address these limitations. Copy number variation sequencing (CNV-seq) and chromosome microarray analysis (CMA) are incapable of identifying balanced chromosomal structural rearrangements or indicating the genomic localization and orientation of duplicated segments or insertions (Riggs et al., 2020; Hu et al., 2023). In this context, optical genome mapping (OGM) using Bionano genome imaging involves the visualization of very long linear single DNA molecules (median size larger than 250 kb) that have been labeled through specific sequence motifs. This technique integrates microfluidics, high-resolution microscopy, and automated image analysis to enable high-throughput whole-genome imaging and *de novo* assembly, thereby providing a significant advancement in identifying the origin and orientation of long DNA molecules (megabase in length). The structural variants pipeline of OGM compares the labeling patterns and inter-label distances between the constructed genome maps of the tested sample and a reference genome, while the copy number variation pipeline of OGM enables the detection of large unbalanced aberrations (typically larger than 5 Mb). This technology is equivalent to an ultra-high-resolution karyotype, achieving approximately 10, 000 times higher resolution than traditional G-banding karyoptye (Mantere et al., 2021). OGM facilitates genome-wide detection of structural variants, including chromosome insertions, deletions, inversions, duplications, and translocations as small as a few hundred base pairs, making it an accurate and comprehensive method for recognizing cryptic BCRs with non-centromeric breakpoints (Mantere et al., 2021; Zhang et al., 2023). Additionally, long-read sequencing, which typically generates reads several kilobases in length, provides a robust approach for characterizing diverse structural variants. It offers unique advantages in investigating regions of the human genome that are challenging to analyze with short-read sequencing, such as highly repetitive or homologous regions (Eisfeldt et al., 2024). Long-read sequencing is promising in uncovering the hidden complexities of chromosomal rearrangements in couples experiencing infertility or recurrent pregnancy failure (Eisfeldt et al., 2024; Watson et al., 2022). However, the accuracy of long-read sequencing remains limited and is still under development (Warburton and Sebra, 2023), thus its practical application in PGT requires further validation. Furthermore, chromosome conformation-based karyotyping (C-MoKa), utilizing three-fold whole-genome sequencing data, demonstrates superior sensitivity in diagnosing intricate rearrangements. It achieves higher fragment resolution (<500 kb) and more precise breakpoint identification (>100 kb) for structural variants (Bao et al., 2024). However, even after diagnosing cryptic BCRs, challenges remain in achieving genetic testing of CNVs smaller than 1 Mb in biopsied samples from embryos. A possible approach is locating the breakpoints and treating them as unique monogenic variants. By applying the workflow of PGT-M, it is possible to determine which chromosomes, either wild-type or derivative, each embryo has inherited.

## Clinical application of PGT-M and challenges

To reduce the possible impact of allele drop-out (ADO) in polymerase chain reaction (PCR)-based direct detection of pathogenic variants using WGA product, single nucleotide polymorphism (SNP) arrays or next-generation sequencing (NGS)-based haplotyping analysis is simultaneous employed. These methods help to discriminate the parental high-risk haplotype (carrying the familial pathogenic variant) and wild-type haplotype (without the familial pathogenic variant) by analyzing genetic markers flanking the gene or locus of interest (ESHRE PGT-M Working Group et al., 2020; Sermon, 2017). Embryonic genetic diagnosis is achieved through the mutual validation of direct pathogenic variant detection and haplotyping analysis. In cases involving intragenic deletions or dynamic mutations, where direct detection of the variants is not always feasible using WGA products, haplotyping analysis serves as the critical point to obtain embryo diagnosis. For this reason, couples and relevant family members with known genetic status are required to identify and select genetic markers located close to the gene or locus of interest during the preclinical work-up of PGT-M (ESHRE PGT-M Working Group et al., 2020). When a *de novo* pathogenic variant is identified in one partner, distinguishing between high-risk and low-risk haplotypes and establishing phasing can vary individually. If the *de novo* variant is in the male partner, phasing can be established through single sperm analysis (ESHRE PGT-M Working Group et al., 2020; Huang et al., 2022). Conversely, if the *de novo* variant is in the female partner, phasing can be deduced using long-read sequencing, and analysis from PBs is also an option (ESHRE PGT-M Working Group et al., 2020; Tsuiko et al., 2023; Peng et al., 2023). Confirming high-risk and low-risk haplotypes before initiating clinical PGT cycles is highly recommended. In certain cases, establishing haplotypes and phasing may rely on the genotypes of the embryos. This approach assumes the presence of at least one affected embryo and one unaffected embryo to accurately determine the phase and identify recombination events. Besides, after PGT-M testing, embryos undergo PGT-A to access chromosome abnormalities before embryo transfer. The clinical outcomes of PGT-M are a critical aspect concerned by both couples and healthcare providers in pre-test counselling. A systemic review analyzing pooled data from 5, 305 PGT-M cycles and 5, 229 embryo transfers reported live birth rates of 29.7% (95%CI 28.5%–31.0%) per IVF cycle and 21.9% (95%CI: 20.8%–23.1%) per embryo transfer (Poulton et al., 2025).

Furthermore, for couples with indications for PGT-M, there may already have patients in their family, or they may be identified as high-risk couples through reproductive genetic carrier screening. A carrier screening-PGT approach serves as a primary prevention strategy for birth defects, enabling the recognition of at-risk couples before conception. Discussions about carrier screening gradually be integrated into preconception counselling for couples planning to have a child (Mei and Platt, 2024). In cases where high-risk couples carrying disease-causing genetic variants on an autosome or the wife carries a disease-causing genetic variant on a sex chromosome, it may not always be possible to predict the severity of the condition in affected offspring because no affected individuals with the corresponding monogenic disease have been observed in the family. Nevertheless, every individual has the right to make informed reproductive choices, such as PGT technologies, which can help prevent the transmission of specific genetic disorders through embryo diagnosis and selective transfer. This approach avoids the possibility of induced abortion due to positive prenatal testing results and ultimately reduces the disease burden on families and society. However, several factors should be carefully considered when opting for PGT. These include the high financial cost, the uncertainty about the availability of transferrable embryos, the inability to predict long-term prognosis of the affected offspring, and the potential risks associated with PGT, such as the possibility of misdiagnosis and the risks of embryo biopsy. The extent of each couple would benefit from PGT remains unclear and varies on a case-by-case basis. In some instances, couples choosing PGT for multiple monogenic diseases may end up with fewer embryos available for transfer, though this approach may potentially reduce the risk of having a child with specific monogenic diseases. Balancing these considerations requires thorough pre-testing genetic counseling, allowing couples to make informed decisions that align with their reproductive goals and values.

## PGT for HLA

Human leukocyte antigens (HLAs) are tissue antigens that play crucial roles in the human immune system. Transplantation of hematopoietic stem cells (HSCs) from HLA-identical donors, free of related disease-causing mutations when required, is the standard treatment for genetic diseases affecting the hematopoietic system (e.g., β-thalassaemia, sickle cell anemia), acquired diseases impacting the immune system (e.g., leukemia), as well as some rare metabolic diseases (e.g., adrenoleukodystrophy) (Shenfield et al., 2005; Tur-Kaspa and Jeelani, 2015). PGT can be used to select embryos that are unaffected by genetic diseases and have HLA matching for siblings (with a probability of 3/16 for autosomal recessive inheritance and 1/8 for X-linked inheritance), or embryos that are HLA-identical in cases of acquired diseases (with a probability of 1/4) (Verlinsky et al., 2004; Van de Velde et al., 2009). HSCs can be collected from cord blood at birth and used for transplantation to the affected sibling, or bone marrow transplantation may subsequently be required if cord blood is insufficient. Theoretically, the HLA locus is complex, highly polymorphic, and carries an additional risk of recombination within a 4 Mb region on chromosome 6. Although PGT-HLA is technically challenging, it is feasible (Rechitsky et al., 2015; Wang et al., 2020), and both general and specific considerations should be fully taken into account. Since HLA typing is a non-pathologic condition, the selection of HLA-matching embryos raises serious concerns involving medical, psychological, ethical, financial, and technical issues. It must also be considered whether the couple truly desires another child or merely needs a new child to cure their affected child. Additionally, how to handle embryos that are not HLA-compatible must be addressed, including the appropriate disposition of these embryos (Shenfield et al., 2005; De Rycke et al., 2020). PGT for HLA matching should fully consider its inherent limitations and ethical issues, including the time period required from the decision-making to transplantation treatment; the relatively higher number of embryos needed to achieve an unaffected live birth with HLA-matching; the potential misdiagnosis rate of genetic testing in PGT; and the variability in success rates of HSC transplantation (Shenfield et al., 2005).

## Special cases in PGT-M

Germline mosaicism refers to the presence of both normal and mutated gametes in one individual. Studies have revealed that the incidence of mosaic mutations in the parental germline is approximately 3.8% based on whole-genome sequencing data (Rahbari et al., 2016). Individuals with germline mosaicism are at an increased risk of having another affected child, even if they themselves are often phenotypically normal. The recurrence risk depends on the proportion of germ cells carrying the mutation (Campbell et al., 2014a; Campbell et al., 2014b). NGS or long-read sequencing based haplotyping strategies have been successfully employed in cases of maternal germline mosaicism, both in families with an affected child and those without affected offspring (Peng et al., 2023; Chen et al., 2023). However, it is worth noting that while male germline mosaicism can be detected through high-depth sequencing of semen samples, female germline mosaicism remains challenging to identify using blood samples alone, as the detection of mosaicism in females often requires more invasive and impractical procedures. For couples with confirmed germline mosaicism, PGT offers a valuable tool to reduce the risk of transmitting the mutation to offspring. Genetic counseling is essential to help couples understand the risks, benefits, and limitations of PGT in the context of germline mosaicism.

## Clinical application of PGT-A and challenges

Aneuploidy is widely recognized as one of the major causes of pregnancy loss (Melo et al., 2023; Dimitriadis et al., 2020). Maternal age is a well-established risk factor for producing aneuploid gametes (Charalambous et al., 2023). Additionally, recent studies have identified variants in several genes involved in the control of chromosome segregation that, although affecting only a small proportion of individuals, may contribute to aneuploidy risk (Sun et al., 2023; Singh et al., 2021). Another clinical concern in reproductive medicine is recurrent implantation failure (RIF), with genetic factors being considered one of the key influencing elements (Franasiak et al., 2021). In these circumstances, PGT-A has been developed as a strategy to improve in-vitro fertilization (IVF) outcomes for couples with advanced maternal age, recurrent miscarriages, and RIF by prioritizing euploid embryos for transfer based on biopsied samples from embryos. Furthermore, evidence suggests that NGS-based PGT-A can enhance pregnancy outcomes for couples with severe male factor (SMF) infertility. Specifically, it has been shown to significantly reduce early miscarriage rates without compromising cumulative ongoing pregnancy rates, making it a viable option for couples with SMF (Xu et al., 2021).

The clinical application of PGT-A remains a topic of significant debate, particularly regarding whether couples can truly benefit from this technology. Despite the controversy, data from the Society for Assisted Reproductive Technology (SART) indicates a substantial increase in the proportion of IVF cycles utilizing PGT, rising from 14% in 2014 to 44% in 2019 in the United States (Bedrick et al., 2022). Studies have shown that the success rates of PGT-A cycles are influenced by maternal age and the number of retrieved eggs. For instance, compared to cycles without PGT-A, the use of PGT-A was associated with a slightly lower cumulative live birth rate (CLBR) in individuals under 35 years of age (67.3% vs 68.6%, RR 0.96; 95% CI 0.93–0.99). Moreover, PGT-A demonstrated higher CLBR in women aged 35–37 years (62.5% vs 59.1%, RR 1.04; 95% CI 1.00–1.08) and 38–40 years (51.3% vs 44.8%, RR 1.14; 95% CI 1.07–1.20) (Harris et al., 2025). Conversely, a retrospective cohort study found that PGT-A was associated with reduced CLBR among patients under 35 years (70.6% vs 71.1%; aOR, 0.82; 95% CI 0.72–0.93) and no significant difference in those aged 35–37 years (66.6% vs 62.5%; aOR, 0.92; 95% CI 0.83–1.01) compared to cycles without PGT-A (Mejia et al., 2022). A multicenter randomized controlled trial reported live birth rates of 77.2% in the PGT-A group (468/606) and 81.8% in the conventional IVF group (496/606) (Yan et al., 2021). A systematic review concluded that there is insufficient high-quality evidence to demonstrate a difference in CLBR, LBR after the first embryo transfer, or miscarriage rate between IVF with and without PGT-A as currently performed (Cornelisse et al., 2020). On the other hand, another systematic review highlighted that PGT-A in patients with recurrent pregnancy failure is associated with improved clinical outcomes, including higher implantation rates, clinical pregnancy rates, ongoing pregnancy rates, and live birth values, as well as lower clinical miscarriage rates compared to conventional IVF/intracytoplasmic sperm injection (ICSI) (Liang et al., 2023). Current low-quality evidence also suggests that PGT-A may enhance LBR per transfer and per patient in cases of unexplained recurrent pregnancy loss (Mumusoglu et al., 2025), well-designed randomized controlled trials comparing ART with PGT-A versus expectant management are still needed to provide more definitive conclusions. It is important to note that some experts argue that the primary purpose of PGT-A is not necessarily to increase CLBR but rather to maximize the chance of live birth per transfer while minimizing the risk of clinical miscarriage, ongoing aneuploid pregnancies, and futile transfers (Seckin and Forman, 2023). When the number of retrieved eggs is fewer than 15, the PGT-A group has been shown to exhibit no significant improvement in CLBR compared to the conventional IVF group (Hu et al., 2024).

The resolution of chromosomal abnormalities in WGA products from embryo biopsy samples, analyzed using array comparative genomic hybridization (aCGH) or NGS-based platforms, is constrained by the empirical resolution established in each laboratory (ESHRE PGT-SR/PGT-A Working Group et al., 2020). For PGT-A using NGS-based platforms, since the aneuploidies are detected through copy-number analysis and normalized across all chromosomes within the sample. This approach can lead to misclassification of genome-wide ploidy abnormalities, such as haploidy or triploidy, as diploid due to the normalization process. Recent advancements have demonstrated the potential for higher resolution in PGT-A. For example, studies have reported NGS-based platforms capable of achieving 1 Mb resolution (Xie et al., 2022), as well as high accuracy in ploidy classification (100%, CI 98.1%–100%) and the identification of microdeletions (99.2%, CI 98.5%–99.8%) using targeted NGS of selected polymorphisms across the genome (Caroselli et al., 2023). Despite these technological improvements, PGT-A remains unable to directly detect CNVs with fragment sizes smaller than 1 Mb. This limitation poses significant challenges, particularly in couples with infertility or RIF, which are crucial to identify possible cryptic translocations. Integrating multiple diagnostic approaches may enhance the accuracy of PGT-A and provide a more comprehensive assessment of chromosomal abnormalities in embryos.

## Mosaic results

The American Society for Reproductive Medicine (ASRM) defines the mosaic in PGT as “presence of more than one chromosomally distinct cell line in a single sample originating from one individual” (Practice Committee and Genetic Counseling Professional Group GCPG of the American Society for Reproductive Medicine, 2020). The rate of mosaicism in PGT embryos varies depending on the developmental stage, with studies reporting mosaicism rates of 15.8%–17.4% in blastocyst (Munné et al., 2019). For embryos that are truly mosaic, three possible testing outcomes should be considered: euploid (false negative), aneuploid (false positive), and mosaic. A false positive result may lead to the mistaken discard of embryos that could result in a healthy live birth, while a false negative result may lead to either no clinical pregnancy or induced abortion due to an affected fetus after embryo transfer. A multicenter study highlighted significant difference in the likelihood of diagnosing mosaicism across providers, ranging from 6.5% to 35.6%. Notably, the overall chance of having at least one euploid blastocyst available for transfer was significantly higher when mosaicism was not reported (Popovic et al., 2024). This variability in diagnosing and interpreting mosaic results across different laboratories raises further concerns about the accuracy and clinical relevance of mosaicism predictions, as some potentially viable embryos will possibly be discarded due to reported mosaicism. While blastocyst biopsy is reliable for detecting whole-chromosome aneuploidies, its ability to accurately diagnose mosaicism remains questionable (Wu et al., 2021). Embryos classified as mosaic exhibit a higher miscarriage rate compared to euploid embryos, prenatal testing indicates that mosaicism often resolves during pregnancies, and infants born after mosaic embryo transfers are generally similar to those from euploid embryo transfers (Viotti et al., 2023).

Clinicians are encouraged to understand the prevalence and reporting structure of mosaic PGT-A results and to track prenatal, perinatal, and pediatric outcomes following the transfer of mosaic embryos (Practice Committees of the American Society for Reproductive Medicine and the Genetic Counseling Professional Group, 2023). Further research is needed to develop test or analysis strategies that can predict the outcomes of transferring mosaic embryos, thereby optimizing clinical decision-making.

## Ethical principle, legal regulations and genetic counseling related to PGT

In the course of IVF, PGT enables prospective parents to select their future offspring based on genetic characteristics. However, a number of ethical issues have emerged in PGT, including its indications (whether it should be limited to disease prevention or expanded to encompass non-medical purposes), the criteria for embryo selection and discard, the clinical management of embryos identified as mosaic or carriers of monogenic disorders, the potential consequences of embryo discard, and privacy concerns regarding genetic information of both parents and offspring (Latham, 2024).

The medical indications for PGT are determined by assessing the estimated risk of couples having a child with known genetic abnormalities and evaluating clinical insights into the interpretation of disease severity. This is achieved by referencing a pre-approved list of genetic conditions, defining disease severity through formal statements, and considering specific factors (Nakasato et al., 2022). Decisions about whether a condition is sufficiently impactful to warrant PGT are highly personal that differ among patients, adding complexity to the ethical landscape. For couples with balanced chromosomal structural abnormalities, except for those with homologous chromosomal translocation, the effectiveness of PGT is well-established. PGT-M was initially developed to identify embryos carrying genes for serious childhood-onset diseases in IVF cycles. Its application for adult-onset monogenic diseases with full penetrance, such as polycystic kidney disease and Huntington’s disease, or predispositions to cancer, such as breast cancer associated with BRCA1 and BRCA2 variants, is generally considered ethically justifiable (Ethics Committee of the American Society for Reproductive Medicine, 2024). However, the use of PGT for monogenic disorders or genomic diseases with incomplete or low penetrance remains controversial. Polygenic embryo screening, relies on statistical modelling, simulations, and sibling pair analyses to predict risk reduction (Capalbo et al., 2024). However, due to the limited number of embryos available for screening and the uncertain accuracy of risk estimates, the actual risk reductions may below expectations for one or more diseases. Accordingly, the ethical appropriateness of using PGT for polygenic risk scores depends on specific circumstances and requires additional consideration (Siermann et al., 2024; Makrythanasis et al., 2023). When PGT is applied for more than one condition (also referred to as combination-PGT) in couples, it has been argued that the criteria for severity established by public legislation or guidelines could be appropriately adjusted for secondary conditions in couples who already have an indication for PGT-M or PGT-SR. Meanwhile, professionals may be more likely to face requests to transfer embryos known to be affected by a condition identified in combination-PGT, which is considered acceptable according to ESHRE guidance (van der Schoot et al., 2019). The expected benefit of PGT for couples with recurrent miscarriage, advanced maternal age, recurrent implantation failure, or severe male factor infertility requires further evidence from additional data.

As for embryo selection, ethical issues arise in monogenic disease carrier embryos with an autosome recessive inheritance pattern or an X-linked inheritance pattern. Specifically, how to handle embryos identified as carriers and ensure couples are fully informed of the related risks to their offspring must be addressed. Additionally, if couples are unwilling to transfer these embryos, their appropriate disposition should be determined. Besides, the selection of HLA-matching embryos also raises serious concerns involving ethical issues, the appropriate disposition of embryos that are not HLA-compatible should be addressed. Broader ethical concerns include the potential for unnecessary IVF treatments, the increasing demand for “designer babies”, the possible embryo abandonment, and unequal access to medical services (Capalbo et al., 2024). Such issues highlight the need for a balanced genetic counseling approach that considers not only the medical and technical aspects of PGT but also its ethical, social, and psychological dimensions to ensure that couples make informed and ethically sound decisions.

Significantly, legislative mandates related to PGT should also be considered. Regulatory approaches for the application of PGT vary across countries, as they may rely on public ordering (statutes or legislation), private ordering (guidelines or self-regulation), or a combination of both (Ginoza and Isasi, 2020). PGT is not permitted in some European countries, and there are also differences in the applications of PGT-M, PGT-SR and PGT-A (European IVF-Monitoring Consortium EIM for the European Society of Human Reproduction and Embryology ESHRE et al., 2024). As reported in the literature, in countries where PGT is permitted, most countries impose some degree of restriction on the clinical application of PGT. These restrictions typically include requirements that PGT be performed only in specifically licensed institutions, regulations specifying which patients and conditions qualify for PGT, as well as guidelines detailing how the PGT process is to be provided and prohibition of nonmedical sex selection (Ginoza and Isasi, 2020). Thus, the clinical application of PGT should comply with national regulations.

Genetic counseling from a genetic counselor with expertise in PGT should be provided throughout the entire PGT process. All couples considering PGT should receive comprehensive and thorough pre-test counselling to help them make informed decisions regarding using PGT in their hereditary conditions, as well as the accuracy of genetic testing and associated technical risks. For individuals with cognitive impairment resulting from monogenic diseases, providing nondirective counselling and make them fully understand the risks, benefits, and limitations of PGT presents tough challenges. Genetic counselling should also be provided to the couple to explain the genetic testing results of biopsied embryos, the limitations of the test, and the necessity of prenatal diagnosis following embryo transfer and clinical pregnancy, thereby facilitating informed decision-making regarding elective embryo transfer.

## The implantation potential of euploid blastocyst in PGT

Even with PGT, the LBR per euploid embryo transfer has been reported at 54.1%–55.1%, despite the great efforts made to improve it (Harris et al., 2025). Successful implantation of a euploid embryo requires not only chromosomal normality but also an adequately thick, immunologically tolerant, decidualized, and receptive endometrium within the window of implantation (Cimadomo et al., 2023). However, many euploid blastocysts fail to implant or result in biochemical pregnancies or miscarriages. The causes of these negative outcomes likely involve a combination of embryonic, maternal, paternal, clinical, and laboratory factors, which remain poorly understood and constitute a “black box” in reproductive medicine (Cimadomo et al., 2023). Recent studies have focused on the embryo-endometrial dialogue, but no statistically significant differences in live births were observed between patients with or without endometrial receptivity analysis before euploid single frozen embryo transfer (44.6% vs 51.3%; adjusted OR 0.87; 95% CI, 0.73–1.04) (Craciunas et al., 2019; Doyle et al., 2022). The ESHRE Time-Lapse Technology Group has recommended combining PGT-A with morphokinetic analysis to enhance the elective transfer of embryos with the highest implantation potential (ESHRE Working group on Time-lapse technology et al., 2020). This suggests that integrating artificial intelligence and non-invasive analytical approaches could further refine this technology into a comprehensive embryo diagnosis and selection modality. Additionally, DNA and RNA sequencing of blastocyst biopsy samples has shown that transcriptomic analysis of euploid embryos can provide valuable insights into their implantation potential, thus offering a promising approach for optimizing selective embryo (Jin et al., 2024). Further academic research is essential to elucidate the endometrial characteristics associated with reproductive fitness, refine, thereby enhancing our understanding of implantation failure and ultimately improving outcomes for couples undergoing PGT.

## The safety of PGT

An additional critical consideration is the safety of embryo biopsy, along with the short- and long-term implications of PGT. Current evidence suggests that obstetric, neonatal, and early childhood outcomes have been reassuring thus far (Practice Committees of the American Society for Reproductive Medicine and the Society for Assisted Reproductive Technology, 2024). A systemic review based on large observational evidence indicated that blastocyst biopsy–the predominant method employed in PGT–did not alter the risk of obstetrical or neonatal outcomes when compared to conventional IVF or ICSI without PGT (Mao et al., 2024; Sites et al., 2021). While the incidences of obstetric and neonatal complications, as well as other adverse events, were found to be comparable between PGT and conventional IVF groups among couples with normal karyotype (Yan et al., 2021), a review study highlighted an elevated incidence of preterm deliveries, birth defects, and pregnancy-related hypertensive disorders associated with trophectoderm biopsy (Alteri et al., 2023). Moreover, offspring conceived through PGT exhibited a higher risk of preterm birth compared to those conceived spontaneously (Ginström Ernstad et al., 2023). Nonetheless, the long-term risk of embryo biopsy still needs more well-designed research to provide evidence.

## Advancements and new insights in PGT

There is a report that preimplantation DNA methylation screening (PIMS) can simultaneously provide information on copy number variations (CNVs) and global DNA methylation levels (Li et al., 2017). Further studies indicate that embryo DNA methylation levels affect the clinical outcomes of ART in both younger women and those of advanced maternal age (≥38 years old); euploid embryos with specific methylation states (level closest to 0.25–0.27) show better live birth rates (Gao et al., 2023). Additionally, a PIMS artificial intelligence (AI) model has been introduced to predict the likelihood of live birth and facilitate elective embryo transfer (Zhan et al., 2023). Another study demonstrates the feasibility of RNA-based PGT by utilizing the abundant mRNA transcript copies in trophectoderm cells to diagnose genetic mutations while simultaneously assessing embryo competence, achieving a significantly higher accuracy rate (up to 95% for direct mutation detection) compared to DNA-based methods (Wang et al., 2024).

As a primary prevention measure, PGT can undoubtedly assist couples in reducing birth defects under specific conditions, following a synthetic evaluation of indications, maternal age, couple fertility, accuracy of embryo detection, and related ethical and/or legal constraints. The technology of PGT will continue to evolve, becoming increasingly comprehensive and precise. However, this technology should be applied in strict compliance with legislation and ethical principles, with the ultimate aim of benefiting couples. Long-term follow-up data on the safety of embryo biopsy, as well as maternal and fetal outcomes, needed to be thoroughly investigated.

## Discussion

PGT plays a pivotal role in the primary prevention of birth defects caused by aneuploidies, chromosomal abnormalities, and monogenic diseases with known pathogenic variants. Particularly for couples with balanced chromosomal structural abnormalities or those indicated for PGT for monogenic disorders, the effectiveness of PGT is well-established. However, the efficacy of PGT in couples with normal karyotype analysis who choose PGT due to recurrent miscarriage, advanced maternal age, or recurrent implantation failure remains questionable. Due to the complexity of genetic materials and the limited quantity of biopsied samples, PGT faces significant technical and ethical challenges. Firstly, regarding technical limitations, overcoming the inherent limitations of WGA approaches remains a critical issue. Secondly, improving the ability to identify complex or cryptic structures of the BCR in couples with infertility, recurrent miscarriage, or recurrent implantation failure is crucial for pre-testing evaluation of PGT indications and enhancing the genetic testing of embryos, particularly for copy number variations (CNVs) involving fragments smaller than 1 Mb. Thirdly, in the context of PGT-M, there are challenges involving the inability of long-read sequencing to resolve haplotypes for specific genomic sequences and the difficulty in determining whether micro-duplications with fragment sizes smaller than 1 Mb are *in situ* (occurring in their original genomic location). These technical limitations highlight the urgent need for continued innovation and refinement of genetic technologies to expand diagnostic capabilities and enhance the accuracy and scope of PGT. Regarding ethical challenges, PGT should be clinically applied in strict compliance with legislation and ethical principles under the circumstance that clear indications exist, with the ultimate aim of benefiting couples. If PGT it to be provided for couples affected by genetic diseases and who have cognitive impairment, or if it could be expanded to include genetic diseases with incomplete or low penetrance, as well as polygenic embryo screening, this remains controversial. More importantly, criteria for embryo selection and discard, as well as the clinically appropriate disposition of embryos identified as mosaic or carriers of monogenic diseases, should be taken into full account before initiating PGT. Genetic counseling should be provided to couples prior to PGT to explain the possible benefits and limitations of embryo biopsy and genetic testing, along with comprehensive and thorough genetic counselling regarding the genetic results of embryos, to help them make informed decisions about the use of PGT and elective embryo transfer. Additionally, further research is needed to evaluate the risk of transferring embryos with mosaic chromosome abnormalities, the implantation potential of euploid embryos, and the long-term health outcomes of children born following PGT.
